# Vitamin D Deficiency in Patients with Diabetes in French Guiana: Epidemiology and Relation with Microvascular and Macrovascular Complications

**DOI:** 10.3390/nu13124302

**Published:** 2021-11-28

**Authors:** Elise Girard, Mathieu Nacher, John Bukasa-Kakamba, Aniza Fahrasmane, Antoine Adenis, Mickael Massicard, Kinan Drak Alsibai, Bertrand De Toffol, Raissa Bekima, Liliane Thelusme, Diana Okambabelle, Magalie Demar, Jean M. Aurelus, Nadia Sabbah

**Affiliations:** 1 Cayenne Hospital Center, Department of Endocrinology and Metabolic Diseases, 97306 Cayenne, French Guiana; elise.girard@ch-cayenne.com (E.G.); john.bukasakakamba@ch-cayenne.fr (J.B.-K.); mickael.massicard@ch.cayenne.fr (M.M.); raissa.bekima@ch-cayenne.fr (R.B.); liliane.thelusme@ch-cayenne.fr (L.T.); diana.okambabelle@ch-cayenne.fr (D.O.); jean.aurelus@ch-cayenne.fr (J.M.A.); 2 Clinical Investigation Center Antilles French Guiana (CIC INSERM 1424), Cayenne Hospital Center, 97306 Cayenne, French Guiana; mathieu.nacher@ch-cayenne.fr (M.N.); aniza.fahrasmane@ch-cayenne.fr (A.F.); antoine.adenis@ch-cayenne.fr (A.A.); 3 Department of Pathology and Center of Biological Resources (CRB Amazonie), Cayenne Hospital Center, 97306 Cayenne, French Guiana; kdrak.alsibai@doctor.com; 4 Department of Neurology Cayenne Hospital Center, 97306 Cayenne, French Guiana; bertrand.detoffold@ch-cayenne.fr; 5 Laboratory of Parasitology-Mycology (LHUPM), Cayenne Hospital Center, 97306 Cayenne, French Guiana; magalie.demar@ch-cayenne.fr; 6 EA3593, Amazon Ecosystems and Tropical Diseases, University of Guiana, 97300 Cayenne, French Guiana

**Keywords:** vitamin D deficiency, diabetes complications, French Guiana, angina pectoris

## Abstract

Vitamin D (VD) insufficiency is common among patients with diabetes in French Guiana. The study aimed to evaluate the prevalence of VD deficiency in the different type of diabetes encountered and to analyze the relationship between VD deficiency and diabetes complications. Methods: An observational study was conducted between May 2019 and May 2020 in French Guiana, based on data from the CODIAM study (Diabetes Cohort in French Amazonia), describing the characteristics of patients with diabetes mellitus. Among 600 patients enrolled with diabetes, 361 had an available VD assay. Results: The mean 25(OH)VD (hydroxycalciferol) level was 27.9 ng/mL. The level of VD was inversely proportional to the HbA1c (glycated hemoglobin) level. Patients with angina pectoris had a greater proportion of deficiencies VD < 20 ng/mL than those without angina. By contrast, patients with retinopathy had higher vitamin D concentrations than those without retinopathy. There was no association between vitamin D and arteriopathy, stroke, nephropathy and polyneuropathy. VD deficiency was more frequent in women, and in patients with a high school education. Conclusion: The prevalence of VD deficiency was high in patients with diabetes in French Guiana, emphasizing the importance of VD supplementation.

## 1. Introduction

Vitamin D (VD) insufficiency is currently defined as a blood level below 30 ng/mL, and VD deficiency is defined as a level below 20 ng/mL according to the World Health Organization (WHO) (2003) and the Endocrine Society (2011) [1,2]. The role of VD deficiency in metabolic pathologies including obesity and diabetes is increasingly discussed [3]. People with darker skin have a higher risk of VD deficiency [4]. In the French Caribbean, 42.6% of the population have a VD below 20 ng/mL, and 69.6% have a VD deficiency, despite year-round tropical sun exposure [5]. Polymorphisms in the VD transporter protein (BPVD) partly explain the differences in (hydroxycalciferol) (25(OH)VD) concentrations between dark and light-skinned populations, while bioavailable VD would remain almost identical for homozygous patients [6]. It has been suggested that several environmental factors such as inadequate diet, lack of sun exposure, or dark phototype may promote VD deficiency [7].

The discovery of VD receptors in the wall of the myocardium and vessels has led to a great deal of work on cardiovascular pathologies, particularly in hypertension and atherosclerosis [8]. The elevation of parathyroid hormone, which is often present in VD deficiency, is associated with cardiovascular risk factors, particularly obesity [9] and diabetes [10].

VD plays a role in insulin resistance, firstly, by stimulating the insulin receptor, and secondly, by acting on the parathyroid hormone and thus indirectly promoting dephosphorylation of the GLUT4 transporter (which diffuses circulating glucose) and by increasing the peroxisome proliferator-activated receptor delta (PPAR delta) gene, which is involved in fat cell accumulation and fatty acid oxidation [11,12,13]. There are sex-specific effects of VD in the pathogenesis of type 2 diabetes [14].

Regarding the microvascular complications of diabetes, VD deficiency exacerbates renal failure, whereas correction of VD deficiency decreases proteinuria in nephropathic patients [15]. VD deficiency contributes to the development of left ventricular myocardial dysfunction in patients with type 2 diabetes and in individuals without a history of coronary heart disease [16]. An intervention study evaluating the effects of VD supplementation on cardiovascular disease markers among African-American and Hispanic type 2 diabetic communities further suggested that the positive effect of VD supplementation on lipid profiles may be mediated by other cofactors related to VD metabolism [17]. Finally, from a social point of view, VD deficiency is more frequent in poor or illiterate populations [18]. The results of current recommendations vary and reflect the disparate contexts, making their applicability uncertain across populations [19].

Moreover, the diet plays an important role in the intake of VD. The population of French Guiana is composed mainly of Creole and immigrants from neighboring Latin America countries. Thus, as in low income countries, the precariousness is very strong in our territory [20], associated with poorly diversified diet (starch or roots with few fruits and vegetables, and exceptional oily fish) [21], and numerous micronutrient deficiencies [22]. These eating habits do not vary according to the seasons of the year because the French Guiana territory have an equatorial climate.

The prevalence of diabetes in French Guiana in 2014 was 10% among the adult population [23] and over 20% among persons over 45 years of age [24]. The CODIAM cohort (Cohort of Diabetes in French Amazonia) is a prospective cohort conducted in French Guiana describing the epidemiological, clinical and biological aspects of the different types of diabetes in French Guiana.

Given the high prevalence of diabetes in French Guiana and the potential role of VD deficiency in the pathophysiology of diabetes or its complications, the main objective of our study was to analyze and describe VD deficiency in patients with diabetes according to the type of diabetes in the CODIAM cohort between May 2019 and May 2020. Two significant thresholds were analyzed: a deficit below 30 ng/mL and a deficiency below 20 ng/mL. The secondary objective was to analyze and describe VD status in patients with macroangiopathic and microangiopathic complications.

## 2. Materials and Methods

Study design and study area: this monocentric study was cross-sectional and was carried out at the André Rosemon Hospital in Cayenne, French Guiana, in the Endocrinology–Diabetology–Nutrition Department, between May 2019 and May 2020.

Climate and sunshine data in French Guiana: In French Guiana, the equatorial climate includes two seasons, the dry season from July to mid-December and the rainy season from mid-December to June, this is however interrupted in March by about a month of return to the dry season [25]; the temperatures remain consistently high around 30 °C with a minimum of 23 °C. The degree of sunshine is quite stable and there are 3 to 4 weeks in the year when it is lower. The climate and sunshine data in 2019 and 2020 were almost similar (Appendix A and Appendix B).

Sampling: The study population was derived from a subset of 361 out of 600 patients with diabetes in the CODIAM cohort with available VD results. Diabetes was defined as an increase in fasting plasma or capillary blood glucose greater than or equal to 1.26 g/L (or 7 mmol/L) on two occasions and/or a blood glucose level taken at any time of the day greater than or equal to 2 g/L. An HbA1c (glycated hemoglobin) level of 6.5% (48 mmol/mol) or higher was also diagnostic.

Selection criteria: inclusion criteria were age >18 years, a confirmed diagnosis of diabetes, and signed informed consent.

Non-inclusion criteria: Excluded were minors, any person refusing to participate in the study, patients under medical care outside of French Guiana, any diagnosis of gestational diabetes, vulnerable persons (protected adults, adults unable to express their consent and not under protective supervision), persons deprived of liberty, and persons hospitalized without consent.

Data collection: Patients were included (first measure-V1) during consultations in the diabetes department of the Cayenne hospital between May 2019 and May 2020. They were also recruited during hospitalizations in one of the hospital departments in Cayenne, during a request for a diabetology opinion. During their follow-up at the weekday hospital or day hospital. medical data (characteristics, medical and surgical history, examination data and latest biological results, including baseline diabetes monitoring parameters) of the patient were collected, as well as sociodemographic data.

End point criteria: The primary end point of our study was the description of the distribution of VD deficiency, defined as 25(OH)VD less than 30 ng/mL, and VD deficiency, defined as 25(OH)VD less than 20 ng/mL among the five types of diabetes studied. These were type 1 diabetes, type 2 diabetes, slow type 1 diabetes, Mody diabetes, and atypical or secondary diabetes.

The secondary endpoint was the analysis of VD deficiency and deficiency among diabetic patients with diabetes-related complications. The diagnosis of complications was made according to the criteria below:

-Retinopathy: 0. no retinopathy; 1. minimal non-proliferative retinopathy; 2. moderate non-proliferative retinopathy; 3. severe non-proliferative retinopathy (preproliferative retinopathy); 4. proliferative retinopathy.

-Nephropathy, presence of microalbuminuria >30 mg/24 h with or without decreased creatinine clearance.

-Neuropathy: presence of sensory or motor disorders on clinical examination, without any other etiology to explain it.

-Arteriopathy: presence of suggestive clinical symptoms with arterial damage on echodoppler and/or pathological systolic pressure index (<0.9).

-Coronaropathy: presence of suggestive clinical symptoms and/or positive stress test and confirmation by coronary angiography or notion of treatment by stent or bypass.

Statistical analysis: Statistical analysis was performed using STATA software^®^ (STATA-CORP^®^, College Station, TX, USA). A descriptive analysis of the study population and clinical data related to five different types of diabetes and VD deficiency was performed. Quantitative data are expressed as the mean and standard deviation for continuous variables and prevalence data were expressed as frequencies and percentages. Comparisons of quantitative variables between groups were made using parametric or non-parametric tests where appropriate. Percentages were compared using Chi-squared tests. The significance level was 5%. The relation of demographic variables and VD status as well as the patients’ VD status and diabetic nephropathy was investigated using logistic regression, and odds ratios and 95% CI (confidence intervals) are presented.

Regulatory and ethical aspects: All included patients were informed of the use of their anonymized data for research. In accordance with the French Data Protection Act and the General Data Protection Regulation, the data processing has been subject to a data protection impact analysis, a registration in the hospital’s data processing register, and a declaration of compliance with MR003. The protocol was approved by the Comité de Protection des Personnes Sud-Est de Clermont-Ferrand (Nos Ref: 2020/CE 05). All patients provided written informed consent.

## 3. Results

### 3.1. Description of the Study Population

Of the 600 previously identified patients with diabetes in the CODIAM cohort, 361 had VD concentration measurement at the first V1 inclusion visit. Five types of diabetes were analyzed in relation to VD status: type 1 diabetes (19 patients, or 5.26% of the sample), type 2 diabetes (317 patients, or 87.8%), slow type 1 diabetes (1 patient, or 0.28% of the sample), Mody diabetes (2 patients, or 0.55%), and atypical (mostly ketosis-prone diabetes) or secondary diabetes (22 patients, or 6.09% of the sample). The distribution of VD deficiency in the five types of diabetes was studied. There was no significant difference between type of diabetes and VD deficiency (Table 1). The mean duration of diabetes was 10.48 years, with a mean HbA1c of 12.6% and a VD level of 27.98 ± 17.4 ng/mL. Regardless of the type of diabetes, 239 patients had a VD level below 30 ng/mL, (66.48% of the sample), with a mean VD level of 21.58 ± 5.72 ng/mL. Overall, pooling all types of diabetes, 97 patients, (26.87%) were VD-deficient with a level below 20 ng/mL. The baseline characteristics of the study population are summarized in Table 1.

There was no significant age difference between the three categories of VD threshold. Diabetic patients with VD < 30 ng/mL had a mean age of 54.2 years (±14.3) versus 57.36 years (±13) for non-deficient patients (*p* = 0.04).

Overall, 121 patients (33.52%) had VD levels > 30 ng/mL. Among the 239 VD-deficient patients (66.48%), 147 were women (61.25%) and 93 were men (38.75%). Female patients were more likely to be deficient than were male patients (72% vs. 59.2%, *p* = 0.011 for VD deficiency < 30 ng/mL and 30.9% vs. 21.7%, *p* = 0.05 for VD deficiency < 20 ng/mL). Vitamin-D-deficient patients spoke mostly Guianese Creole and Haitian Creole. These two communities alone accounted for 48.95% of the deficient patients among the nine mother tongue languages encountered. Diabetic patients on ALD (long-term conditions) insurance schemes were mostly VD deficient, as were patients under the CMU (Couverture maladie universelle (universal health coverage) scheme.

Counterintuitively, those with fewer years of schooling were less likely to be VD-deficient. This remained significant after adjusting using logistic regression for age, sex, and body mass index for VD < 20 ng/mL, with an adjusted odd ratio (aOR) of 3.4 (95% CI 1.7–6.7) for those with education above high school and for VD < 30 ng/mL, an aOR of 2.6 (95% CI 1.2–5.9) for those with education above high school.

The mean body mass index (BMI) among the VD-deficient patients was slightly higher (31.9 ± 8.4), than in the non-deficient patients (30.1 ± 5.5), but this failed to reach statistical significance (*p* = 0.055)

**Figure 1:**
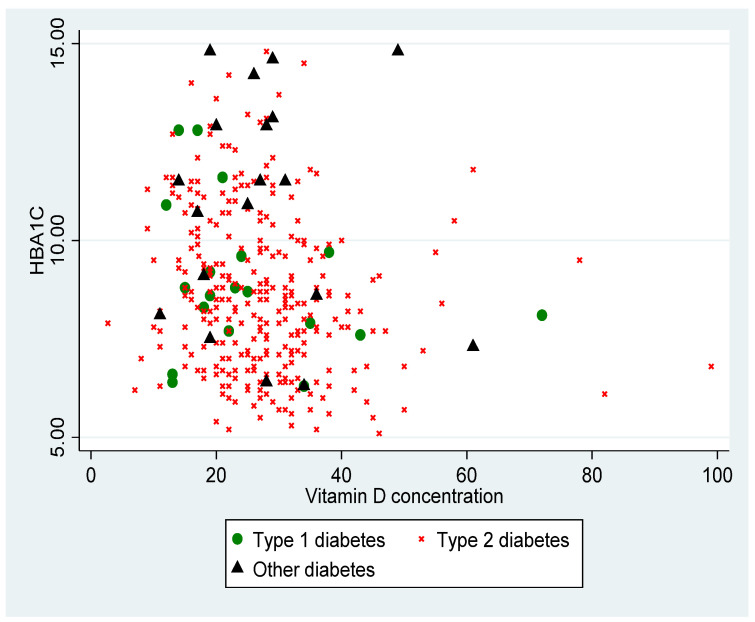
Distribution of HbA1c (%) according to VD (vitamin D) level in ng/mL, according to type of diabetes (*p* < 0.001).

Figure 1 shows the distribution of the HbA1c level according to the VD level, according to the different types of diabetes. The VD level was inversely proportional to the HbA1c (*p* < 0.001).

### 3.2. Results of Complications amongst VD-Deficient Diabetic Patients

For macroangiopathies, 5.22% of patients had a history of transient ischemic stroke, 7.6% of angina pectoris, and 12.5% of arteriopathy. For microangiopathies, 11.36% had retinopathy, 16% had nephropathy, and 14.6% had polyneuritis.

1. Macroangiopathies (Table 2)

Among those with available information, the prevalence of arteriopathy was not significantly different between patients with a VD deficiency whatever the deficit. VD deficiency below 20 ng/mL was more frequent in the group of patients with a history of coronary artery disease (9/19 (47.4%) vs. 55/224 (24.5%), *p* = 0.03). However, there were no significant differences for the 30 ng/mL threshold. The mean VD level in patients with a history of angina was 23.33 ng/mL. There was no significant relationship between history of transient ischemic stroke and vitamin D insufficiency or deficiency in our study.

After adjustment for native language, we found no significant difference between VD-deficient and non-deficient patients for any type of diabetes for the 30 ng/mL threshold or the 20 ng/mL threshold.

2. Microangiopathies (Table 2)

There was no significant relation between nephropathy and vitamin D deficiency. After adjusting for the covariates of high blood pressure, LDL cholesterol, BMI, and smoking, there was still no relation between vitamin D concentration and nephropathy (Table 3). However, patients with diabetic retinopathy had significantly greater median vitamin D concentrations than those without retinopathy (respectively, 31 ng/mL (interquartile range (IQR) = 23–34) vs. 26 ng/mL (IQR = 23–32), *p* = 0.03).

For diabetic neuropathy, there was no significant relation between vitamin D concentration and polyneuropathy.

## 4. Discussion

Our study is the first concerning the evaluation of VD status among patients with diabetes in French Guiana and was performed for two different VD thresholds: 30 ng/mL and 20 ng/mL. Our work indicates that there was a significant proportion of patients with diabetes with VD deficiency in French Guiana, regardless of the type of diabetes encountered. The mean VD in this population was around 27.98 ng/mL. VD deficiency is more frequent in darker-skinned ethnic groups. This has also been shown in other studies in the Caribbean, notably in Guadeloupe [5,6]. Indeed, African-Americans have been found to be at greater risk of VD deficiency than Caucasians [26]. Skin pigmentation influences the efficiency of epidermal synthesis of vitamin D3 (VD3), by competition with melanin, which absorbs UVB photons responsible for the photolysis of 7-dehydrocholesterol into pre-VD3 [27]. In our study, the majority of our patients were French Guianese with type 2 diabetes, with a predominance of women, as described elsewhere [23].

Regardless of the VD deficiency threshold considered (30 ng/mL or 20 ng/mL), there was no difference in the proportion of patients with vitamin D deficiency between the five types of diabetes studied. The two communities most represented were people born in French Guiana and people from Haiti, who are mostly of African descent and hence at greater risk of vitamin D deficiency [28].

Women were more likely to be vitamin D-deficient than men, as observed elsewhere [29]. Besides exhibiting a higher rate of deficiency, women are also at greater risk of developing osteoporotic complications [30]. It was unexpected and counterintuitive observe that, after adjustment for age, sex, and BMI, those with more years of education were more at risk of VD deficiency; a possible hypothesis is that those with more education are less likely to be exposed to sunlight—because they have white-collar jobs—than those who have fewer years of education, who also may need to walk from place to place.

There was no significant correlation between BMI and VD deficiency, but the BMI of patients with deficits tended to be higher than that of patients without vitamin D deficiency. Some authors have hypothesized that, in obese patients, the sequestration of VD in adipose tissue decreases the bioavailability of fat-soluble VD3 [31].

We observed an inverse correlation between HbA1c level and VD concentration, which has previously been described in the literature. A recent Brazilian cohort showed that patients with good glycemic control had higher VD levels [32]. A study on more than 6000 people with diabetes also found a strong association between low HbA1c level and high VD concentration, also in connection with a decrease in insulin resistance [33]. In particular, we noted a decrease in insulin resistance in diabetic patients treated with VD, and thus an improvement in glycemic control [34,35]. A meta-analysis published in 2019 showed that VD supplementation would improve glycemic control in patients with type 2 diabetes [36].

There was no relation between VD deficiency and polyneuropathy or nephropathy. It has been hypothesized that nephropathy could be worsened by VD deficiency, but we did not find any evidence for this. In the Brazilian cohort the authors found a specific relation between VD and the decrease of albuminuria in patients with type 2 diabetes partly due to glycemic control [32]. Some studies found a link between VD and insulin sensitivity with, in particular, an improvement in the release of insulin at the level of pancreatic beta cells; others found an indirect effect improving insulin release via intracellular calcium modulations [37,38,39]. Several studies –including ours—have not found an obvious link between VD and nephropathy and some describe the absence of improvement of renal function with VD treatment [40,41]. Concerning neuropathy, several studies have found an association between VD deficiency and neuropathy or diabetic foot [42,43], but a study by Niu et al. only describes this association for people over 65 years of age; reflecting the youth French Guiana’s demographics, the average age of our cohort was 55 years, which could explain the absence of a link [44].

By contrast, for retinopathy, there was a significantly higher vitamin D concentration among those with retinopathy than among those without. Our findings are in contrast with other studies in the literature.

VD deficiency has been described by some teams as associated with early thinning of the retinal nerve layer with an inversely proportional relation between diabetic retinopathy and active VD3 level [45,46]. Other studies did not show a significant association between diabetic retinopathy and maculopathy according to VD status [47].

Cardiovascular disease remains the leading cause of death in most patients with diabetes mellitus. Nevertheless, we found no relation between vitamin D concentration and arteriopathy or stroke. However, those with vitamin D concentrations below 20 ng/mL were significantly more likely to have a history of angina pectoris. A correlation between low serum 25(OH)VD concentrations and major cardiovascular risks has been reported in other prospective observational studies. Several mechanisms may explain this association: regulation of the renin-angiotensin system, which affects cardiac contractility, vascular tone, and, indirectly, calcification and proliferation of vascular muscle cells [48,49]. VD deficiency is associated with an atherogenic lipid profile [2], and one study suggested that adding VD status to the Framingham risk score could improve the assessment of cardiovascular risk factors in patients with diabetes [50]. However, the association between status and VD and certain cardiometabolic disorders remains uncertain in the scientific community, as shown by some intervention trials, where VD supplementation had no beneficial clinical effect [51,52].

Most of studies do not describe the patients’ sun exposure, nor their diet or eating habits, which may influence the level of 25(OH)VID3. One previous study revealed that 25(OH)VITD2 level was not influenced by the seasons [53]. However, the authors did not find an association between VD deficiency and retinopathy or macrovascular complications [53].

Without non-diabetic controls, it was impossible to judge whether the decrease in VD was a risk factor for diabetes. Some authors suggested that the VD metabolite ratio was a better indicator of VD status, however, we did not evaluate this criterion [54]. Another limitation is that our sample was hospital-based and perhaps not representative of the population of patients with diabetes followed by private practitioners. It was also a cross-sectional study, thus limiting conclusions about the direction of the reported associations. We do not have details on the dietary intake of the patients included in the study, but they are mainly immigrants, with a diet rich in starch and poor in fatty fish, fruits and vegetables [22]. Finally, the number of patients with micro- or macroangiopathic complications may have been insufficient to detect more subtle statistical differences or may have led to some associations that were due to chance. Nevertheless, the present study provides precise estimates of the prevalence of vitamin D deficiency among patients with diabetes and identified risk factors in French Guiana and hence will be useful to focus medical attention on the problem. Future studies on larger samples of this new cohort will further analyze the relation between vitamin D levels and incidence of micro- and macroangiopathic complications.

## 5. Conclusions

Our study revealed a major VD deficiency affecting about 66.48% of patients with diabetes (mainly type 2) in French Guiana. This problem remains underestimated because of the lack of guidelines and because different ethnic groups may have different definitions of vitamin D deficiency. In our study, patients with retinopathy had greater vitamin D concentrations than those without retinopathy and, on the contrary, those with angina pectoris had lower vitamin D concentrations than those without it. VD concentrations were negatively correlated with HbA1c concentration. Overall, as reflected by the discrepancies in the literature, there were no clear relations between VD and diabetes’ microvascular and macrovascular complications and no clear insights as to how to better manage patients with diabetes. VD dosing for certain at risk populations such as those with diabetes should nevertheless be encouraged in French Guiana given the high frequency of VD deficiency. The question of supplementation requires randomized controlled studies to control for potential confounders.

## Figures and Tables

**Table 1:** Characteristics of the sample population according to vitamin D (vitamin D) level regardless of diabetes type, expressed as number “No.” of patients and percentages (%); age, BMI and duration of diabetes expressed as mean and standard deviation SD.

	VD < 20 ng/mL	*p* Value	VD [20–30] ng/mL	*p* Value	VD > 30 ng/mL
VARIABLES			No. and %		
Overall population	97 (26.87)		143 (39.61)		121 (33.52)
Gender		*p* = 0.05		*p* = 0.01	
Women	63 (30.88)		84 (41.18)		57 (27.94)
Men	34 (21.66)		59 (37.58)		64 (50.39)
Age, mean(SD)	54.73 (14.85)		53.91 (14.04)		57.36 (13.00)
BMI, mean (SD)	32.45 (10.34)		31.44 (6.98)		30.17 (5.53)
Duration of diabetes mean (SD)	11.71 (10.82)		9.67 (10.17)		10.48 (9.23)
School		*p* = 0.01		*p* = 0.007	
<Highschool	39 (20.74)		78 (41.49)		71 (37.77)
≥Highschool	43 (36.13)		42 (35.29)		34 (28.57)
Social security		*p* = 0.04		*p* = 0.002	
None	2 (40.0)		3 (60.0)		0 (0)
AME State insurance	2 (9.52)		6 (28.57)		13 (60.90)
CMU welfare	9 (16.07)		22 (39.29)		25 (44.64)
ALD insurance	74 (29.72)		101 (40.56)		74 (29.72)
Mother tongue		*p* = 0.19		*p* = 0.21	
French	15 (24.59)		24 (39.34)		22 (36.07)
Guianese creole	31 (33.70)		38 (41.30)		23 (25.0)
Haitian creole	14 (16.09)		34 (39.08)		39 (44.83)
Portuguese	7 (24.14)		14 (48.28)		8 (27.59)
Spanish	4 (30.77)		4 (30.77)		5 (38.46)
English	8 (32.0)		10 (40.0)		7 (28.0)
Dutch	2 (66.67)		1 (33.33)		0 (0)
Antilles creole	5 (35.71)		5 (35.71)		4 (28.57)
Other	10 (28.57)		13 (37.14)		12 (34.29)
Diabetes type		*p* = 0.28		*p* = 0.63	
Type 1	9 (47.37)		5 (26.32)		5 (26.32)
Type 2	80 (25.24)		129 (40.69)		108 (34.07)
Type slow 1	0 (0)		0 (0)		1 (100.0)
Mody type	1 (50)		0 (0)		1 (100.0)
Atypical	7 (33.33)		9 (42.86)		6 (28.57)
Diabetes total	97 (26.87)		143 (39.61)		121 (33.52)

CMU: Couverture Maladie Universelle (universal health coverage; low-income patients (French or legal residents) receiving social insurance benefits), AME (aide medical d’état) is an emergency assistance for undocumented foreigtients without health insurance.

**Table 2:** Distribution of the number (No.) of patients by diabetes-related microvascular and macrovascular complications according to vitamin D threshold.

	VD < 20 ng/mL	*p* Value	VD [20–30] ng/mL	*p* Value	VD > 30 ng/mL	Total
Microvascular complications			No. (%)			
Retinopathy *YES*	8 (19.51)	*p = 0.52*	11 (26.83)	*p = 0.01*	22 (53.66)	41
*NO*	60 (27.65)		90 (41.47)		67 (30.88)	217
*unknown*	29 (28.16)		42 (40.78)		32 (31.07)	103
Nephropathy *YES*	16 (27.59)	*p = 0.99*	13 (22.41)	*p = 0.01*	29 (50.0)	58
*NO*	68 (26.77)		107 (42.13)		79 (31.1)	254
*unknown*	13 (26.53)		23 (46.94)		13 (26.53)	49
Polyneuropathy *YES*	17 (32.08)	*p = 0.36*	11 (20.75)	*p = 0.07*	25 (47.17)	53
*NO*	70 (25.09)		122 (43.73)		87 (31.18)	279
*unknown*	10 (34.48)		10 (34.48)		9 (31.03)	29
Macrovascular complications			No, (%)			
Arteriopathy *YES*	13 (28.89)	*p = 0.4*	14 (31.11)	*p = 0.42*	18 (40.0)	45
*NO*	60 (23.53)		109 (42.74)		86 (33.73)	255
*unknown*	24 (39.34)		20 (32.79)		17 (27.87)	61
Angina *YES*	9 (47.37)	*p = 0.09*	5 (26.31)	*p = 0.85*	5 (26.32)	19
*NO*	55 (24.55)		97 (43.30)		72 (32.14)	224
*unknown*	2 (28.57)		3 (42.86)		2 (28.57)	7
TIS *YES*	2 (15.38)	*p = 0.66*	7 (53.85)	*p = 0.54*	4 30.77)	13
*NO*	61 (26.75)		95 (41.67)		72 (31.58)	228
*unknown*	2 (25.0)		2 (25.0)		4 (50.0)	8

TIS (transient ischemic stroke).

**Table 3:** Logistic regression investigating diabetic nephropathy for the threshold of vitamin D, vitamin D < 30 ng/mL, and five confounders. BMI, body mass index.

Nephropathy	Odd Ratio	95% CI	*p* Value
Vitamin D < 30 ng/mL	1.539	0.591–4.007	0.3
Arterial hypertension	1.28	0.468–3.498	0.6
Low density lipoprotein cholesterol (LDL)	1.011	0.624–1.639	0.9
BMI	1.013	0.971–1.056	0.5
Currently smoking	0.292	0.579–1.470	0.1
Age	1.068	1.029–1.108	0

## Data Availability

The data sets used and analyzed during the current study available from the corresponding author on reasonable request.

## Appendix A

Temperatures, precipitations and sunshine averages in French Guiana between 1991 and 2010. https://meteofrance.fr/sites/meteofrance.fr/files/files/editorial/BilanClimatique2020_OM_FINAL.pdf (accessed on 21 November 2021).

## Appendix B

The sunshine averages in French Guiana in 2020 compared to the period between 1996 and 2014 (https://meteofrance.gf/fr/climat/bulletin-climatique-annuel-2020) (accessed on 21 November 2021).

The yellow columns correspond to the average sunshine in 2020, and the grey columns correspond to the to the average sunshine during the period 1996–2014.

Janv = January; Fév = February; Mars = March; Avril = April; Mai = May; Juin = June; Juil = July; Août = August; Sept = September; Oct = October; Nov = November; Déc = Deccember.

## Appendix C

Daily sunshine in French Guiana in May 2019. http://www.meteo.fr/temps/domtom/antilles/packpublic/alaune/bcm/DernierBCMOM_973.pdf (accessed on 21 November 2021).
